# Predictive value of matrix metalloprotease 9 on surgical outcomes after pericardiectomy

**DOI:** 10.1186/s13019-022-01796-9

**Published:** 2022-03-23

**Authors:** Likui Fang, Wenfeng Yu, Guocan Yu, Bo Ye, Gang Chen

**Affiliations:** Department of Thoracic Surgery, Affiliated Hangzhou Chest Hospital, Zhejiang University School of Medicine, Hangzhou, 310003 China

**Keywords:** MMP9, Constrictive pericarditis, Postoperative outcomes

## Abstract

**Background:**

The effects of matrix metalloproteases (MMPs) and tissue inhibitors of metalloproteinases (TIMPs) expressions on the patients with constrictive pericarditis undergoing pericardiectomy remain unclear. This study explored the associations of MMPs and TIMPs expressions with postoperative outcomes in these patients.

**Methods:**

Pericardial specimens were obtained during pericardiectomy from the patients with constrictive pericarditis. The levels of MMP1, MMP2, MMP9 and TIMP1 in pericardium were analyzed by quantitative real-time polymerase chain reaction. The enrolled patients were divided into two groups according to the optimal cutoff value of gene expression predicting postoperative complications. Postoperative outcomes were compared between the two groups. Binary logistic regression analysis was performed to determine the degree of contribution of gene expression on postoperative outcomes.

**Results:**

A total of 22 patients and their pericardial specimens were included. The level of MMP9 was significantly associated with postoperative complications and the optimal cutoff value predicting postoperative complications was 3.67. The patients with low level of MMP9 (< 3.67) had lower incidence of postoperative complications (*P* = 0.002), shorter postoperative intensive care unit (*P* = 0.040) and hospital stay (*P* = 0.043) in comparison to those with high level of MMP9 (≥ 3.67). Binary logistic regression analysis showed that high level of MMP9 increased the risk of postoperative complications (OR 27.096, 95% CI 1.166–629.886, *P* = 0.040).

**Conclusions:**

High level of MMP9 in the pericardium was associated with poor postoperative outcomes and was the independent risk factor of postoperative complications. The level of MMP9 could be used as a potential marker for prediction of surgical outcomes.

**Supplementary Information:**

The online version contains supplementary material available at 10.1186/s13019-022-01796-9.

## Introduction

Constrictive pericarditis is a rare disease caused by pericardial inflammation, fibrosis and inelasticity [1]. The etiology of constrictive pericarditis is various in different areas. Most cases in Europe and North America are idiopathic or related to prior cardiac surgery or chest irradiation, while tuberculosis is the most common cause in developing counties [2, 3]. Constrictive pericarditis is chronic and progressive in most cases, and leads to diastolic heart failure with poor quality of life and prognosis [4]. The treatment approaches of constrictive pericarditis are limited. Palliative treatment such as diuretic therapy only reduces symptoms temporarily in chronic cases, and surgical pericardiectomy is the only definitive treatment to relieve the pericardial constriction [5, 6]. However, pericardiectomy is associated with high incidence of postoperative complications and in-hospital mortality [7, 8].

Matrix metalloproteases (MMPs) are a large family of zinc dependent endopeptidases that can degrade almost every component of the extracellular matrix (ECM), and the enzymatic activity of MMPs can be blocked by tissue inhibitors of metalloproteinases (TIMPs) through binding to the active site of MMPs [9]. Abnormal expressions of MMPs and TIMPs are involved in a variety of pathological conditions, including inflammation and fibrosis [10]. In addition, the levels of MMPs have been reported to be associated with surgical outcomes and could be potential biomarkers predicting postoperative complications [11–13]. However, the roles of MMPs and TIMPs in the patients with constrictive pericarditis undergoing pericardiectomy have not been investigated. This study aimed to explore the effects of MMPs and TIMPs expressions on postoperative outcomes in those patients.

## Methods

### Study population

The pericardial specimens were obtained during pericardiectomy from the patients with constrictive pericarditis in our department between January 2018 and November 2019. The tissue specimens were collected from the pericardium over the left ventricle and the size was 0.5 cm by 0.5 cm. A total of 22 specimens were successfully collected from the patients. We retrospectively reviewed the records of these patients and their characteristics including demographic, preoperative and outcomes data were extracted from the hospital electronic medical records system. The study protocol was approved by the Institutional Review Board of Affiliated Hangzhou Chest Hospital, Zhejiang University School of Medicine (ID of ethics approval: 20160830) and written patient informed consent was obtained.

### Interventions and outcomes

The preoperative diagnosis of constrictive pericarditis mainly depended on the clinical symptoms, echocardiography, chest enhanced computed tomography and central venous pressure (CVP). Pericardiectomy was routinely performed by median sternotomy in all patients without the use of cardiopulmonary bypass. The extent of pericardiectomy included at least the anterolateral pericardium between the two phrenic nerves, the basal pericardium over the diaphragmatic surface, the pericardium on the great arteries and the pericardium from superior vena cava-right atrium junction to inferior vena cava-right atrium junction [14].

The primary outcome was the incidence of postoperative complications which were defined as the comorbidities that occurred after surgery but did not exist before. Second outcomes were postoperative intensive care unit (ICU) stay, postoperative hospital stay and in-hospital mortality.

### Specimens analysis

The expressions of MMP1, MMP2, MMP9 and TIMP1 mRNA were analyzed by quantitative real-time polymerase chain reaction (qRT-PCR) using Hieff UNICON® qPCR SYBR Green Master Mix (YISHEHG, Shanghai, China). GADPH mRNA was utilized as the endogenous control. Total RNA was isolated using Trizol reagent (Invitrogen) and complementary DNA was synthesized using Hifair® II 1st Strand cDNA Synthesis Kit (YISHEHG, Shanghai, China). Specific primers used for qRT-PCR assays were 5′-GGGAGATCATCGGGACAACTC-3′, 5′-GGGCCTGGTTGAAAAGCAT-3′ for MMP1; 5′-TGATCTTGACCAGAATACCATCGA-3′, 5′- GGCTTGCGAGGGAAGAAGTT -3′ for MMP2; 5′-GTGCTGGGCTGCTGCTTTGCTG-3′, 5′-GTCGCCCTCAAAGGTTTGGAAT-3′ for MMP9; 5′-CTTCTGGCATCCTGTTGTTG-3′, 5′-AGAAGGCCGTCTGTGGGT-3′ for TIMP1; 5′-TGCACCACCAACTGCTTAGC-3′, 5′-GGCATGGACTGTGGTCATGAG-3′ for GADPH.

### Statistical analysis

The relative expressions of target genes were calculated by ΔΔCT method. The fold change in gene expression was calculated as 2^−ΔΔCT^. The patients were first divided into two groups according to postoperative complications. The measurement data and the enumeration data were statistically analyzed with the Mann–Whitney *U* test and the Fisher exact test, respectively. The receiver operating characteristic (ROC) curve and Youden Index were used to determine the cutoff values of MMPs or TIMPs predicting postoperative complications. Then, the patients were regrouped according to the cutoff value. Binary logistic regression analyses were performed to determine the correlation between MMPs/TIMPs and postoperative complications. Confounders were included, based on univariate analysis. These analyses were conducted using SPSS software (version 24.0, IBM SPSS Inc. United States). Statistical significance was set at *P* value < 0.05 (all P values presented were two-sided).

## Results

### Group division

A total of 22 patients and corresponding pericardial specimens were enrolled in this study. A total of 12 postoperative complications were observed in 10 (45.5%) patients (Additional file 1: Table S1), with one patient dying of cardiac failure on the fifth postoperative day. The most common complication was low cardiac output (7 cases), followed by cardiac failure (2 cases), acute kidney injury (2 cases) and pulmonary embolism (1 case). The patients were classified into two groups according to postoperative complications and we found that the patients with postoperative complications have higher level of MMP9 than those without postoperative complications (*P* = 0.002) (Table 1).

**Table 1:** Expressions of MMPs and TIMP1 in the constrictive pericardium based on the postoperative complications

Variables	Postoperative complications		*P* value
	Yes (N = 10)	No (N = 12)	
MMP1	5.99 (− 0.44–11.79)	9.16 (2.96–13.06)	0.539
MMP2	2.83 (0.75–4.63)	2.00 (0.80–3.05)	0.080
MMP9	5.77 (3.19–11.11)	3.32 (2.04–4.85)	0.002
TIMP1	1.53 (− 1.10–2.98)	0.81 (− 0.54–2.55)	0.180

Values presented as median (range)

MMP, matrix metalloproteinase; TIMP, tissue inhibitors of matrix metalloproteinase

**Fig. 1:**
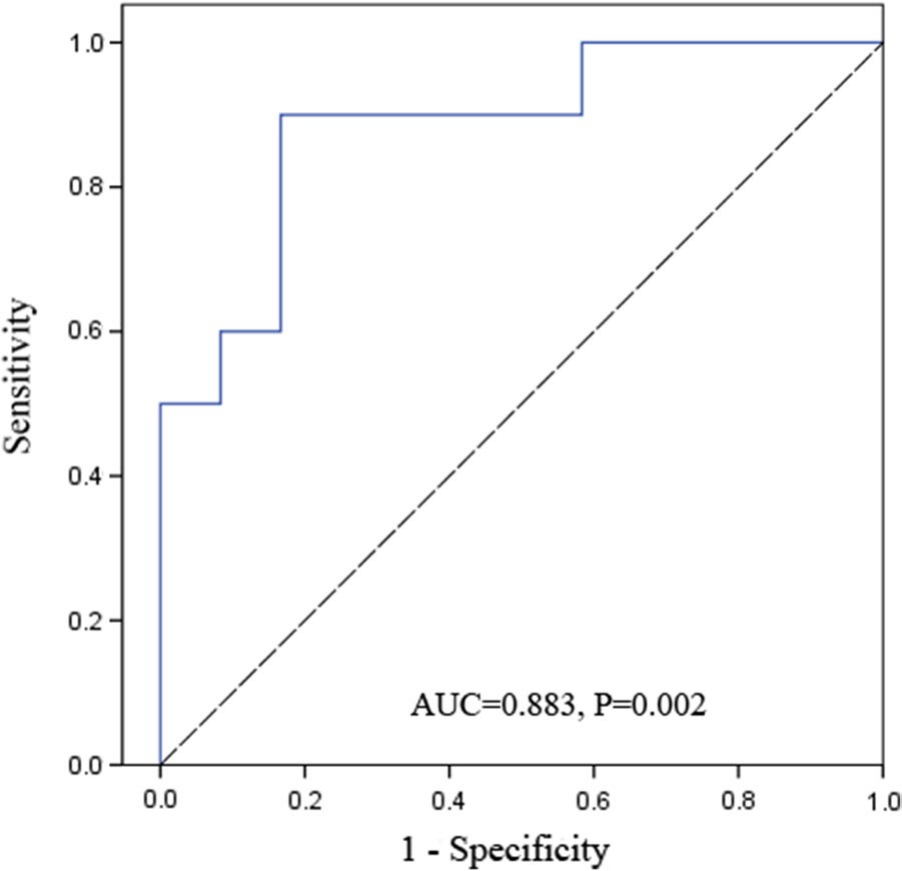
The area under the ROC curve for postoperative complications determined using the level of MMP9. ROC, receiver operating characteristic; AUC, area under the curve

The result of ROC curve also presented the significant correlation between MMP9 and postoperative complications. The area under curve (AUC) was 0.883 (95% CI 0.739–1.000, *P* = 0.002) (Fig. 1). The Youden Index was calculated and the result showed the optimal cutoff value of MMP9 level was 3.67 (sensitivity 90.0%, specificity 83.3%, Youden Index 0.733). According to the cutoff value, the patients were divided into the MMP9-low group (MMP9 < 3.67) and the MMP9-high group (MMP9 ≥ 3.67). There were no statistical differences between the two groups for gender, age, etiology, body mass index, cardiac functional class, preoperative CVP and other baseline characteristics (Table 2).

**Table 2:** Baseline characteristics of study patients based on the level of MMP9 in the constrictive pericardium

Variables	MMP9-low group (N = 11)	MMP9-high group (N = 11)	*P* value
Gender			
Male	11 (100%)	11 (100%)	
Age, years	70 (54–80)	73 (54–80)	0.270
Etiology			–
Tuberculosis	11 (100%)	11 (100%)	
Preoperative NYHA functional class			0.327
I	2 (18.2%)	0 (0%)	
II	1 (9.1%)	3 (27.3%)	
III	8 (72.7%)	8 (72.7%)	
Hypertension	1 (9.1%)	2 (18.2%)	1.000
Diabetes	2 (18.2%)	2 (18.2%)	1.000
Heart disease*	2 (18.2%)	4 (36.4%)	0.635
BMI, kg/m^2^	21.3 (17.3–24.8)	21.0 (17.7–24.8)	0.652
Pulse rate, beats/min	98 (80–112)	90 (80–145)	0.699
Preoperative CVP, cmH_2_O	23.0 (20.5–30.0)	30.0 (20.4–42.5)	0.065
Pericardial thickness, mm	10.7 (8.2–16.0)	10.7 (7.0–16.0)	0.847
LVEF, %	54.3 (50.0–56.4)	56.0 (51.9–64.0)	0.101
CRP, mg/L	11.7 (5.0–21.0)	17.0 (5.0–62.5)	0.076
ESR, mm/h	37.0 (3.0–52.0)	37.0 (17.0–68.0)	0.300

Values presented as N (percentage) for categorical variables and median (range) for continuous variables

MMP9-low was defined as the level of MMP9 < 3.67 and MMP9-high was defined as MMP9 ≥ 3.67

MMP, matrix metalloproteinase; NYHA, New York Heart Association; BMI, body mass index; CVP, central venous pressure; LVEF, left ventricular ejection fraction (measured on echocardiogram); CRP, C-reactive protein; ESR, erythrocyte sedimentation rate

^*^Heart disease included atrial fibrillation and coronary heart disease

### Postoperative outcomes

The comparison of outcomes between the MMP9-low group and the MMP9-high group was shown in Table 3. Compared with the MMP9-high group, the MMP9-low group had significantly lower incidence of postoperative complications (9.1% vs. 81.8%, *P* = 0.002). In addition, the MMP9-low group had shorter postoperative ICU stay (*P* = 0.040) and postoperative hospital stay (*P* = 0.043) in comparison to the MMP9-high group. One in-hospital death occurred in the MMP9-high group with no mortality in the MMP9-low group.

**Table 3:** Postoperative outcomes of study patients stratified by the level of MMP9 in the constrictive pericardium

Variables	MMP9-low group (N = 11)	MMP9-high group (N = 11)	*P* value
Postoperative CVP, cmH_2_O	17.0 (5.0–20.0)	17.8 (5.0–32.0)	0.365
Postoperative intubation, h	11 (3–132)	28 (5–232)	0.173
Duration of using vasoactive agents, h	0 (0–116)	47 (0–143)	0.114
Postoperative complications	1 (9.1%)	9 (81.8%)	0.002
Postoperative ICU stay, days	2 (1–5)	4.5 (1–10)	0.040
Postoperative hospital stay, days	18 (13–24)	23 (13–29)	0.043
In-hospital mortality	0 (0%)	1 (9.1%)	1.000

Values presented as median (range) for continuous variables and N (percentage) for categorical variables

MMP9-low was defined as the level of MMP9 < 3.67 and MMP9-high was defined as MMP9 ≥ 3.67

MMP, matrix metalloproteinase; CVP, central venous pressure; ICU, intensive care unit

### Multivariate analysis

In order to determine the degree of contribution of MMP9 on postoperative outcomes, the statistically significant factors in univariate analysis were included in multivariate regression model (Additional file 1: Table S2). Binary logistic regression analysis demonstrated that compared with the MMP9-low group, the risk for postoperative complications significantly increased in the MMP9-high group (OR 27.096, 95% CI 1.166–629.886, *P* = 0.040) (Table 4).

**Table 4:** Effect of the level of MMP9 in the constrictive pericardium on postoperative outcomes

Groups	Postoperative complications		
	OR	95%CI	*P* value
MMP9-low group	1	–	–
MMP9-high group	27.096	1.166–629.886	0.040

MMP9-low was defined as the level of MMP9 < 3.67 and MMP9-high was defined as MMP9 ≥ 3.67

MMP, matrix metalloproteinase; OR, odds ratio; CI, confidence interval

## Discussion

Surgical pericardiectomy is the curative treatment for constrictive pericarditis and is strongly suggested in the patients with progressive symptoms after medical therapy [15]. Despite being considered effective, pericardiectomy is associated with non-negligible risk of postoperative complications and in-hospital mortality. There have been a number of studies exploring the risk factors of poor outcomes after pericardiectomy, and the results showed that surgical outcomes depended heavily on the functional status of patients, the etiology of constrictive pericarditis, the timing of surgical intervention, the extent of pericardial resection and the need for cardiopulmonary bypass [16–20]. However, most of the studies represented single-center and retrospective experiences. Tzani, A. et al. performed a meta-analysis to systematically review the clinical outcomes of patients undergoing pericardiectomy for constrictive pericarditis [21]. This meta-analysis included 27 eligible studies and 2114 patients. The results highlighted that radiation and after-cardiac surgery patients had a significantly high mortality risk, and that surgical intervention should be considered before advanced symptoms occurred, and that total pericardiectomy without the use cardiopulmonary bypass was preferred.

However, the association between abnormal gene expressions in pericardium and postoperative outcomes has not been investigated. MMPs are well-known mediators of cardiovascular pathophysiology. The changes of MMPs expressions are directly associated with inflammation and the subsequent formation of fibrosis, a key pathological process of many diseases including constrictive pericarditis [22, 23]. This study first evaluated the associations of MMPs expressions in pericardial tissues with postoperative outcomes in patients undergoing pericardiectomy for constrictive pericarditis. We found a positive correlation between the level of MMP9 and postoperative outcomes. The results showed that overexpression of MMP9 could increase the incidence of postoperative complications and prolong the length of postoperative ICU and hospital stay. Multivariate analysis further verified that high level of MMP9 in the pericardium was the independent risk factor of postoperative complications. It was worth mentioning that the predictive value for postoperative complications was significant when the cutoff value of MMP9 level was 3.67, with 90.0% sensitivity and 83.3% specificity.

The major postoperative complication in this study was low cardiac output which mainly resulted from the presence of myocardial fibrosis and atrophy. Previous studies indicated that MMP9 played an important role in tissue remodeling related to cardiac function because of its central role in inflammation and elastin degradation which led to decreased elasticity [23, 24]. In addition, enhanced activation of MMP9 was associated with the state of active myocardial remodeling and could be a potentially useful marker for the identification of patients at risk for heart failure development and poor outcome [25].

There are some limitations that should be pointed out. Firstly, as this is a single-center retrospective study, the selection bias is inevitable. Secondly, the sample size is small, which may interfere in our findings. It is difficult to increase sample size in a small period of time due to the low incidence of constrictive pericarditis. Finally, lacking of normal tissue analysis may also influence our findings. Therefore, further studies with large amounts of samples were required to verify our preliminary results.

## Conclusions

Our study has shown that although effective at relieving symptoms of constrictive pericarditis, pericardiectomy was associated with high incidence of postoperative complications. Increased expression of MMP9 in the pericardium was significantly associated with poor postoperative outcomes and was the independent risk factor of postoperative complications. The level of MMP9 could be used as a potential marker for prediction of surgical outcomes.

## Supplementary Information

**Additional file 1**. Supplemental Table.

## Data Availability

The datasets used during the current study are available from the corresponding author on reasonable request.

## Abbreviations

MMPs: Matrix metalloproteases
TIMPs: Tissue inhibitors of metalloproteinases
CVP: Central venous pressure
ICU: Intensive care unit
qRT-PCR: Quantitative real-time polymerase chain reaction
ROC: Receiver operating characteristic
AUC: Area under curve
