# Supplementary material for: Predictive value of matrix metalloprotease 9 on surgical outcomes after pericardiectomy
Source: J Cardiothorac Surg. 2022 Mar 23;17:50. doi: 10.1186/s13019-022-01796-9 (PMC8943958; doi:10.1186/s13019-022-01796-9)
Supplement: Supplementary file 1 — Additional file 1. Supplemental Table. [file 13019_2022_1796_MOESM1_ESM.docx]

Supplemental Table 1. Postoperative complications after pericardiectomy in study patients

| Complications | N=10 |
| --- | --- |
| Low cardiac output | 7 (70.0%) |
| Septic shock | 2 (20.0%) |
| Acute kidney injury | 2 (20.0%) |
| Pulmonary embolism | 1 (10.0%) |

Values presented as N (percentage).

There were 2 patients suffering two postoperative complications.

Supplemental Table 2. The analysis of perioperative characteristics predicting postoperative complications

| Variables | Postoperative complications | | | P value | Multivariate analysis | | |
| --- | --- | --- | --- | --- | --- | --- | --- |
|  | No (N=12) | | Yes (N=10) |  | OR | 95%CI | P value |
| Gender  Male | 12 (100%) | | 10 (100%) | / |  |  |  |
| Age, >70y | 4 (33.3%) | | 8 (80%) | 0.043 | 6.409 | 0.304-135.100 | 0.232 |
| Etiology |  | |  | / |  |  |  |
| Tuberculosis | | 12 (100%) | 10 (100.0%) |  |  |  |  |
| Preoperative NYHA functional class |  | |  | 0.628 |  |  |  |
| Ⅰ | 2 (16.7%) | | 0 (0%) |  |  |  |  |
| Ⅱ | 2 (16.7%) | | 2 (20.0%) |  |  |  |  |
| Ⅲ | 8 (66.7%) | | 8 (80.0%) |  |  |  |  |
| Hypertension | 2 (16.7%) | | 1 (10.0%) | 1.000 |  |  |  |
| Diabetes | 2 (16.7%) | | 2 (10.0%) | 1.000 |  |  |  |
| Heart disease* | 2 (16.7%) | | 4 (40.0%) | 0.348 |  |  |  |
| BMI, kg/m2 | 21.2 (17.3-24.8) | | 21.1 (17.7-23.5) | 0.539 |  |  |  |
| Pulse rate, beats/min | 95 (85-112) | | 90 (80-145) | 0.539 |  |  |  |
| Preoperative CVP, cmH_2_O | 24.0 (20.5-30.0) | | 31.0 (20.4-42.5) | 0.093 |  |  |  |
| Pericardial thickness, mm | 10.5 (8.2-16.0) | | 10.7 (1.0-15.6) | 0.628 |  |  |  |
| LVEF, % | 55.0 (50.0-56.4) | | 56.0 (51.9-64.0) | 0.254 |  |  |  |
| CRP, mg/L | 11.2 (5.0-21.0) | | 22.0 (11.4-62.5) | 0.004 | 1.220 | 0.873-1.706 | 0.245 |
| ESR, mm/h | 35.5 (3.0-52.0) | | 37.0 (17.0-68.0) | 0.314 |  |  |  |
| Operative duration, min | 225 (200-313) | | 269 (157-380) | 0.381 |  |  |  |
| Blood loss, ml | 100 (100-200) | | 100 (100-700) | 1.000 |  |  |  |
| MMP9 level  <3.67  ≥3.67 | 10 (83.3%)  2 (16.7%) | | 1 (10.0%)  9 (90.0%) | 0.002 | 27.096 | 1.166-629.886 | 0.040 |

Values presented as N (percentage) for categorical variables and median (range) for continuous variables.

OR, odds ratio; CI, confidence interval; NYHA, New York Heart Association; BMI, body mass index; CVP, central venous pressure; LVEF, left ventricular ejection fraction (measured on echocardiogram); CRP, C-reactive protein; ESR, erythrocyte sedimentation rate; BNP, brain natriuretic peptide; MMP, matrix metalloproteinase

* Heart disease included atrial fibrillation and coronary heart disease.
